# Cytogenetic Characterization and Metabolomic Differences of Full-Sib Progenies of *Saccharum* spp.

**DOI:** 10.3390/plants12040810

**Published:** 2023-02-10

**Authors:** Yi Wang, Ru Li, Baoshan Chen

**Affiliations:** 1 State Key Laboratory for Conservation and Utilization of Subtropical Agro-Bioresources, Guangxi University, Nanning 530004, China; 2 College of Horticulture and Landscape Architecture, Zhongkai University of Agriculture and Engineering, Guangzhou 510225, China

**Keywords:** *Saccharum* spp., smut disease, genomic in situ hybridization, metabolomics, disease resistance

## Abstract

Sugarcane smut is a worldwide fungal disease. Disease resistance breeding is the most economical and effective measure to prevent and control sugarcane smut. The cytogenetic characteristics and metabolomic differences of sugarcane F_1_s are closely related to disease resistance. Zhongzhe 1 and G160 sugarcane from the same parents (ROC25 and Yunzhe89-7) were used; the plants were grown in accordance with the barrel method. When the seedlings had 4–5 leaves, genomic in situ hybridization (GISH) was performed; digoxigenin (DIG)-labeled female parental (ROC25)DNA and biotin-labeled male parental (Yunzhe89-7) DNA were used as probes, and the karyotypes of two hybrids were analyzed. The new sugarcane smut-resistant variety (Zhongzhe 1) and the susceptible variety (G160) derived from the same parent were analyzed via gas chromatography—mass spectrometry technology (GC–MS) to compare the metabolomic differences between them. GISH analysis revealed that the chromosome ploidy number of Zhongzhe 1 sugarcane and G160 sugarcane were 114 and 110, respectively. However, the two contain different numbers of chromosomes from the female (ROC25) and male (Yunzhe89-7) parents. Moreover, 258 significantly changed metabolites were identified in smut-resistant Zhongzhe 1, as compared with the smut-susceptible G160 sugarcane: 56 flavonoids, 52 phenolic acids, 30 lipids, 26 organic acids, 26 amino acids and derivatives, 19 nucleotides and derivatives, 5 alkaloids, 9 terpenoids, and 35 others. Multivariate statistical analysis revealed a distinct difference in metabolic pathways between Zhongzhe 1 sugarcane and G160, and both of these varieties had unique functional metabolites. Differences in chromosome composition may constitute the genetic basis for the difference in resistance to smut disease between Zhongzhe 1 sugarcane and G160 sugarcane, and a high accumulation of flavonoids, lipids, terpenoids and tannins may constitute the basis of resistance to smut disease for the Zhongzhe 1 variety.

## 1. Introduction

Sugarcane is a perennial tropical plant that accounts for two-thirds of the world’s sugar production and serves as a feedstock for bioenergy production [1]. Sugarcane smut caused by *Sporisorium scitamineum*, a basidiomycetous fungus, is a worldwide disease of sugarcane, resulting in a 20–50% loss in cane yield in general [2,3,4]. The infected sugarcane plant is characterized by long slender leaves and small stem, and a black whip that emerges from the top of the plant at the late stage of infection [5]. Breeding of disease-resistant varieties has long been the most economic and effective measure to control the smut disease [6,7].

Modern sugarcane varieties are derived from complex interspecific crosses between the domesticated sugarcane species *Saccharum officinarum* L. (2*n* = 80) and the wild species *Saccharum spontaneum* L. (2*n* = 40–128) [7]. Sugarcane varieties or cultivars (*Saccharum* hybrid) currently ground are mostly from the crossing of selected germplasms with desired agronomic traits, such as high yield, high sugar content, and disease resistance [8,9,10]. Genes from a sugarcane smut-resistant variety, for example, may be infiltrated into the genome of individual progenies [11,12]. Unlike diploid plants, F_1_ population of a cross between two sugarcane parents segregates in both phenotype and genotype with varied numbers of chromosomes in the individuals. To corelate the phenotype, karyotype analysis is often used to characterize the chromosomes in the F_1_ progenies [7,13].Karyotype analysis can not only be used to study the genetic variation, systematic evolution and genetic relationship between species but can also be used to inform cross-breeding and breeding [14] and provide a theoretical basis for the identification of hybrid offspring. Understanding chromosome behavior in intergeneric hybrids, such as chromosome elimination and unreduced gametes resulting from different coexisting chromosomes, is important for practical application in introgression programs [15]. Sequential fluorescence *in situ* hybridization (FISH) and genomic *in situ* hybridization (GISH) are techniques that can be used as part of a robust approach for the precise identification of alien chromatin within sugarcane [13,14,15].

In addition to cytogenetic characteristics, metabolomic-related traits of intergeneric hybrids are also important for identifying useful and undesirable genes. The ability of plants to cope with various chemical and physical stressors depends on a number of metabolites [16]. Metabolites play important roles in plant adaptation to chemical and physical stressors; this is especially true for enzymes involved in stress repair and degradation of damaged cellular components, allowing plants to recover from and survive stress [17]. Flavonoids can promote the physiological survival of plants and protect them from fungal pathogens and environmental stress [18,19,20]. Apigenin 7-O-(6″-O-acetylglucoside) and quercetin 3-(2-caffeoylsophoroside) 7-glucoside synthesis was increased during the infection of sugarcane by *S. scitamineum*, becoming an important marker metabolite related to healthy sugarcane plants [21]. Lyso-phosphatidylethanolamine (LPE) belongs to lipid metabolites, and LPE treatment induced the expression of genes involved in the signaling and biosynthesis of the defense-related hormone salicylic acid (SA) [22]. Moreover, phenolic acids and organic acids are some of the most important components involved in plant disease resistance, and the synthesis of phenolic compounds acts as the first defense to protect plants from fungal pathogens [23]. 

In our previous studies, we reported the breeding of new sugarcane varieties Zhongzhe series, progenies of a cross from varieties ROC25 and Yunzhe89-7, that exhibited excellent agronomic traits, including high resistance to the smut in the field [8,9,10]. As a matter of fact, more than 17,000 progeny lines were obtained from the cross of ROC25 and Yunzhe89-7. Sub populations with specific traits, including smut resistant and smut susceptible, were developed from this large population (our unpublished data). The availability of suitable materials offers an opportunity to corelate a specific trait with the chromosomal constitution in a line. In this study, the smut-resistant sugarcane variety Zhongzhe 1 and smut-susceptible line G160, both derived from the same cross (ROC25 × Yunzhe89-7) were used as experimental materials for comparative cytogenetic and metabolic analyses. GISH was performed, during which digoxigenin (DIG)-labeled ROC25 DNA and biotin-labeled male parental (Yunzhe89-7) DNA were used as probes, and the karyotypes of the two hybrids were analyzed based on the GISH analysis results. Moreover, we analyzed the metabolites of Zhongzhe 1 sugarcane and G160 sugarcane to identify metabolites that may be associated with disease resistance. The detailed findings will contribute to sugarcane breeding for smut resistance.

## 2. Results

### 2.1. Chromosome Counting for Identification of Zhongzhe 1 Sugarcane and G160 Sugarcane, Which Are Derived from the Same Parents

**Figure 1:**
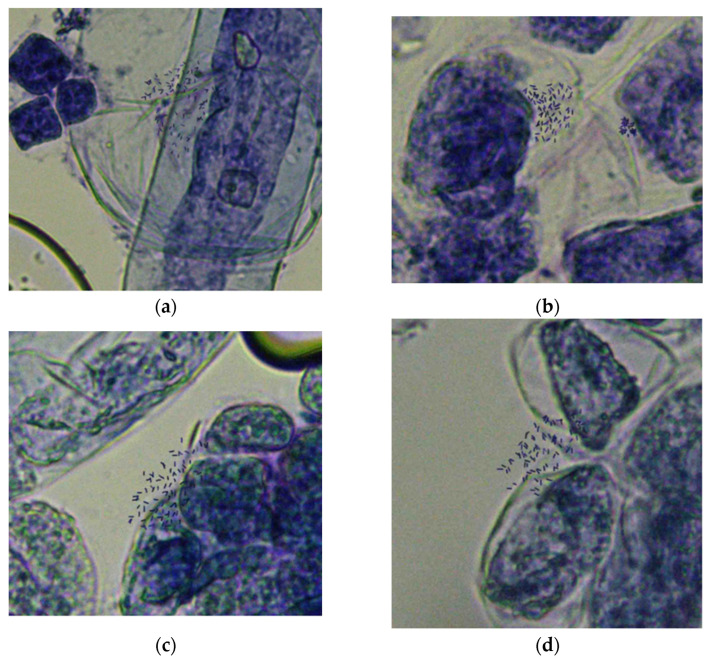
Metaphase chromosomes of ROC25 and Yunzhe89-7 sugarcane varieties and their hybrid offspring. (**a**) ROC25; (**b**) Yunzhe89-7; (**c**) Zhongzhe 1 sugarcane; (**d**) G160.

We obtained chromosome preparations suitable for chromosome counting for the female parent (ROC25), the male parent (Yunzhe89-7), and their hybrid offspring—Zhongzhe 1 sugarcane and G160 sugarcane. The chromosomes were thoroughly spread with little cytoplasm background in all the materials. The chromosome counting results are shown in Figure 1. The modal number of chromosomes for the ROC25, Yunzhe89-7, Zhongzhe 1 sugarcane and G160 sugarcane varieties was 2*n* > 80 and ranged from 80 to 114.

### 2.2. GISH of F_1_ Hybrids Resulting from ROC25 × Yunzhe89-7

**Figure 2:**
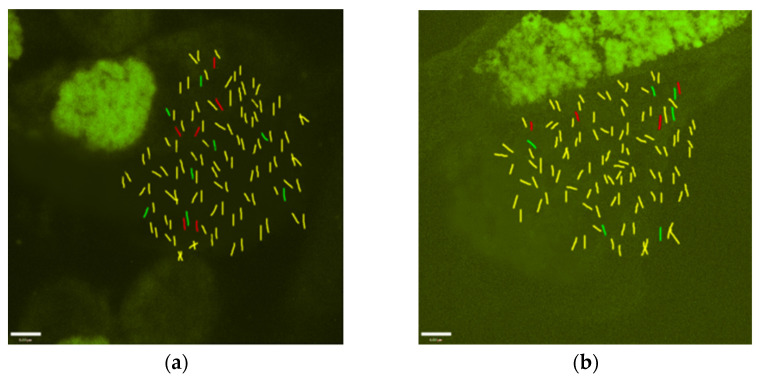
The red color represents the signal of the DIG-labeled female parent (ROC25) DNA probe, and the green color represents the signal of the biotin-labeled male parent (Yunzhe89-7) DNA probe (Bar = 6 µm). (**a**) Zhongzhe 1 sugarcane; (**b**) G160 sugarcane.

GISH was carried out on the metaphase chromosomes of Zhongzhe 1 sugarcane and G160 sugarcane, which are hybrids resulting from a ROC25 × Yunzhe89-7 cross. Among the DNA of the chromosomes of the hybrids, the sequences homologous to those of Yunzhe89-7 fluoresced green, and the sequences homologous to those of ROC25 fluoresced red. The results of GISH confirmed the ploidy levels of Zhongzhe 1 sugarcane and G160 sugarcane (Figure 2), and GISH analysis showed that the chromosome numbers of Zhongzhe 1 sugarcane and G160 sugarcane were 114 and 110, respectively. However, due to the high degree of homology of the ROC25 and Yunzhe89-7 sugarcane genomes, ROC25-derived and Yunzhe89-7-derived chromosomes appeared yellow. The fluorescence of the chromosomes of the two groups differed when their sequences were different, namely, red or green (Figure 2a,b).

### 2.3. Metabolomic Differences between the Hybrids Resulting from ROC25 × Yunzhe89-7

**Figure 3:**
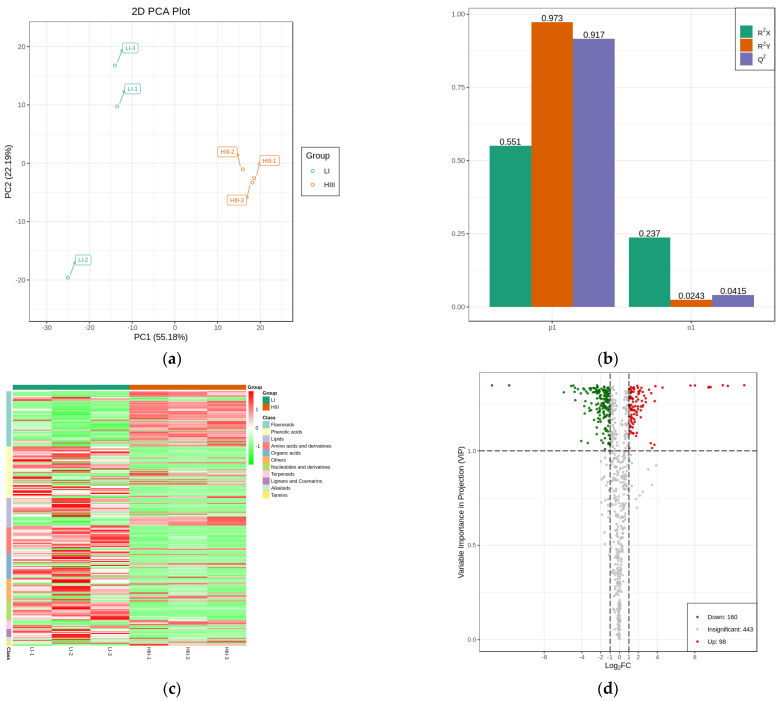
Results of a multivariate statistical analysis of metabolites from Zhongzhe 1 sugarcane, which exhibits high resistance to smut disease (HIII), and G160 sugarcane, which exhibits low resistance to smut disease (LI). (**a**) PCA score plot of metabolites in young shoots of HIII plants (red) and LI (green) plants; the *x*-axis represents the first PC, and the *y*-axis represents the second PC. (**b**) PLS-DA score plot of metabolites between the young shoots of HIII (red) plants and LI (green) plants. (**c**) The 701 metabolites identified were divided into 11 categories. (**d**) Volcano plots of metabolites between the young shoots of HIII plants and LI plants. Metabolites with q values > 0.05 are highlighted in red when they increased in abundance and in green when they decreased in abundance.

Metabolite profiling experiments were performed on the groups to investigate the relationships between plant metabolites and smut disease resistance of Zhongzhe 1 sugarcane, which exhibits high resistance (HIII) to smut disease, and G160 sugarcane, which exhibits low resistance (LI) to smut disease. Principal component score plots of principal component (PC1) vs. PC2 were obtained for the samples (55.18% and 22.19%, respectively), and the total contribution reached 77.37% (Figure 3A). The principal component analysis (PCA) score plots suggested that the metabolite profiles of Zhongzhe 1 sugarcane and G160 sugarcane were markedly different. To compare the differences in metabolites between Zhongzhe 1 sugarcane and G160 sugarcane, partial least-squares discriminant analysis (PLA-DA) models were established under both ionization modes (Figure 3b). The PLS-DA models displayed a strong goodness of fit (R^2^X) and high predictability (Q^2^), which were as 0.551 and 0.917 (Figure 3b), respectively, for the comparison of Zhongzhe 1 sugarcane and G160 sugarcane. The PLS-DA models were validated by response permutation testing (RPT), which revealed the absence of overfitting and no false positives among the experimental data.

A total of 701 metabolites were identified through positive and negative ionization modes based on a self-constructed database (MetWare) and the HMDB (http://www.hmdb.ca/, accessed on 14 September 2021) (Supplementary Table S1). To obtain a clearer overview of the changes in the metabolite concentrations during three developmental stages, a heatmap was plotted for the 701 chemical constituents (Figure 3C); as shown in the figure, the red and green colors represent relatively high and relatively low intensities, respectively. The heatmap revealed that the concentrations of various metabolites, including alkaloids, amino acids, flavonoids, lignans and coumarins, lipids, nucleotides, flavonol and flavonol/flavone glycosides, phenolic acids, organic acids, terpenoids, and their derivatives, were distinctly different between the leaf samples of Zhongzhe 1 sugarcane and G160 sugarcane. Metabolites with variable importance in projection (VIP) scores greater than 1.0 were considered significantly changed metabolites (SCMs) for potentially distinguishing the samples identified via the PLS-DA models. In this study, using a VIP score of greater than 1.0 as the cutoff, we searched for differentially accumulated metabolites between Zhongzhe 1 sugarcane and G160 sugarcane. Metabolites for which P was < 0.05 via Student’s t test were considered statistically significantly differentially accumulated. A total of 258 metabolites were identified as SCMs (Figure 3d, Supplementary Table S2), and the percentage of SCMs in each category was calculated (Table 1). A total of 258 SCMs (|log_2_(FC)| > 1) were classified as flavonoids, lipids, phenolic acids, terpenoids, alkaloids, amino acids and their derivatives, nucleotides and their derivatives, and others (Table 1). Among them, 56 flavonoids significantly differentially accumulated, and flavonoids were the metabolites whose concentration changed the most between the two sugarcane varieties, indicating that there were significant differences in flavonoid accumulation between Zhongzhe 1 sugarcane and G160 sugarcane, whose resistance to sugarcane smut differs. Among the metabolites detected, 44.1% of phenolic acids, 40.1% of flavonoids and 30.1% of lipids were significantly differentially expressed between Zhongzhe 1 sugarcane and G160 sugarcane (Table 1), indicating that, in sugarcane, phenolic acids, flavonoids and lipids may be closely associated with smut disease resistance.

### 2.4. Differentially Enriched Metabolic Pathway Analysis

**Figure 4:**
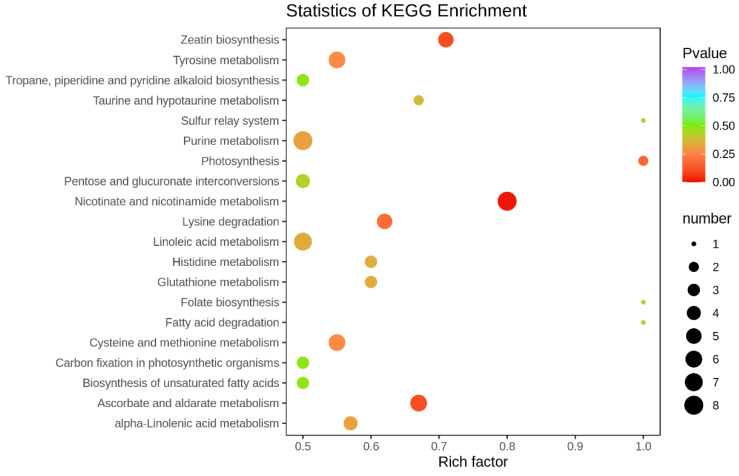
Results of KEGG pathway enrichment analysis. The *x*-axis represents the enrichment factor, and the *y*-axis represents the various pathways. The dot color represents the p value, and the dot size represents the number of differentially accumulated metabolites.

The 258 differentially accumulated metabolites were subjected to KEGG functional enrichment to identify metabolic pathways enriched in more than two different metabolites. Pathway enrichment analysis revealed metabolic pathways that markedly significantly differed. As shown in Figure 4, a comparison of Zhongzhe 1 sugarcane, which exhibits high resistance to smut disease, and G160 sugarcane, which exhibits low resistance to smut disease, revealed that 20 metabolic or biosynthetic pathways were affected. The differentially accumulated metabolites found in Zhongzhe 1 sugarcane and G160 sugarcane are involved primarily in the following metabolic or biosynthetic pathways: zeatin biosynthesis, tyrosine metabolism, photosynthesis, nicotinate and nicotinamide metabolism, cysteine and methionine metabolism, tyrosine metabolism, α-linolenic acid metabolism, purine metabolism, glutathione metabolism, histidine metabolism, histidine metabolism, linoleic acid metabolism, taurine and hypotaurine metabolism, folate biosynthesis, the sulfur relay system, fatty acid degradation and ascorbate and aldarate metabolism (Figure 4). The top four pathways in terms of the p value associated with metabolic pathway enrichment analysis were “nicotinate and nicotinamide metabolism”, “zeatin biosynthesis”, “ascorbate and aldarate metabolism” and “lysine degradation”. Among these metabolic pathways, “nicotinate and nicotinamide metabolism” and “ascorbate and aldarate metabolism” involved more differentially accumulated metabolites than did the other metabolic pathways (8 and 7, respectively).

### 2.5. Analysis of the Validation of Key Genes Involving Metabolites

**Figure 5:**
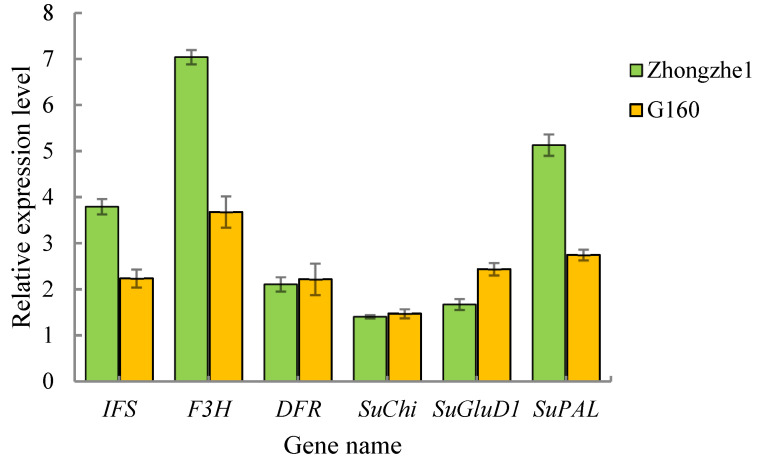
Results of a qRT–PCR analysis of 6 genes that encode key synthesis-related enzymes in the leaves of sugarcane varieties (Zhongzhe 1 sugarcane and G160).

Quantitative real-time PCR (qRT–PCR) was used to compare the relative expression differences of genes that encode key enzymes (isoflavone synthase, *IFS*; flavonoid 3′-hydroxylase, *F3H*; dihydroflavonol 4-reductase, *DFR*) involved in the synthesis of flavonoids and three other important genes that encode enzymes involved with resistance-related metabolites (chalconeisomerase, *SuChi*; Glutamate dehydrogenase 1, *SuGluD1*; phenylalanineammonia-lyase, *SuPAL*) in Zhongzhe 1 sugarcane and G160 sugarcane (Figure 5). The qRT–PCR results showed that the expression levels of the genes that encode key enzymes involved in the synthesis of flavonoids were consistent with the trends of the relative contents of flavonoids in Zhongzhe 1 sugarcane and G160 sugarcane. The expression levels of three important genes involved in defense-related metabolites were consistent with the metabolomic data in Zhongzhe 1 sugarcane and G160 sugarcane. These results indicated that the measured metabolomic data are valid and reliable.

## 3. Discussion

Sugarcane is a heterozygous polyploid or aneuploid plant species with a very complex genetic background [24,25]. During cross-breeding in sugarcane, chromosomes are inherited in various forms due to the unbalanced distribution of haploid chromosomes [7,26]. Due to the high homology of the sugarcane ROC25 sugarcane genome and the Yunzhe89-7 sugarcane genome, ROC25-derived and Yunzhe89-7-derived chromosomes appeared yellow. The fluorescence of the two groups of chromosomes differed when their sequences were different—red or green. GISH analysis showed that the ploidy number of chromosomes in G160 sugarcane was 110, of which four were from the female parent (ROC25) and six were from the male parent (Yunzhe89-7); these appeared as red or green when their sequences were different. Moreover, GISH analysis showed that the ploidy number of Zhongzhe 1 sugarcane was 114, of which six chromosomes were from the female parent (ROC25) and eight were from the male parent (Yunzhe89-7); these appeared as red or green. Differences in chromosome composition may constitute the genetic basis for the differences in resistance of Zhongzhe 1 sugarcane and G160 sugarcane to smut disease.

**Figure 6:**
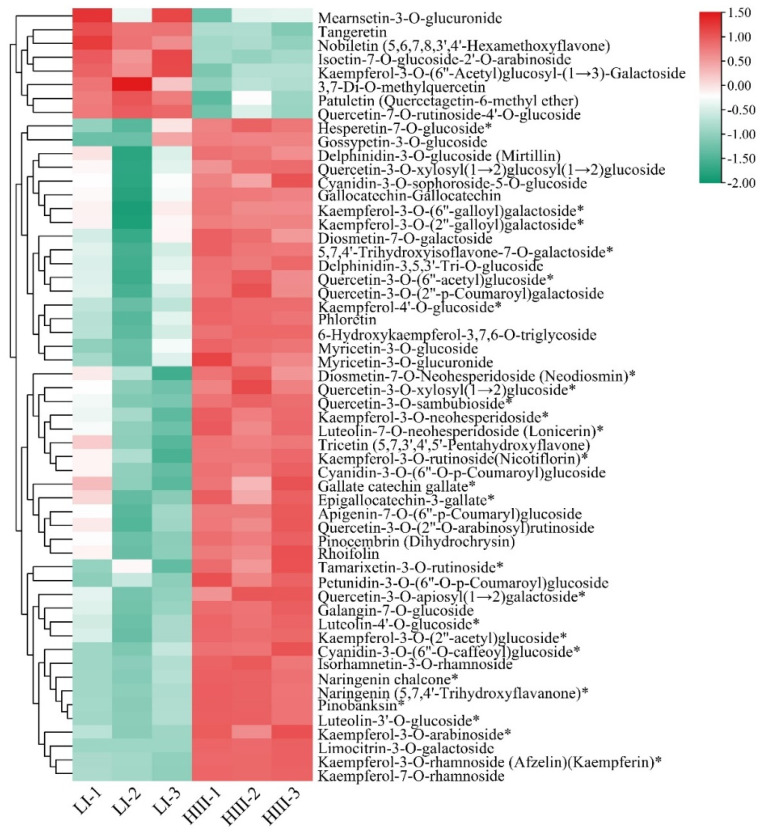
Fifty-six flavonoids that differentially accumulated between Zhongzhe 1 sugarcane and G160 sugarcane. * indicates the existence of isomers of the compound.

In addition to cytogenetic characteristics, metabolomic-related traits of intergeneric hybrids are important in identifying useful and undesirable genes. Several studies have reported differences in terms of morphology, physiology and disease resistance of Zhongzhe 1 sugarcane and G160 sugarcane hybrids, whose resistance to smut disease differs and which results from ROC25 × Yunzhe89-7 [10], but little is known about the molecular basis of these differences. Metabolites play important roles in plant adaptations to chemical and physical stressors; this is especially true for enzymes involved in stress repair and degradation of damaged cellular components, as these enzymes allow plants to recover from and survive stress [17]. Metabolomics, which has high throughput, accuracy, sensitivity, and repeatability, is enabling rapid advancements in molecular biology [27,28]. In the present study, a total of 258 differentially accumulated metabolites were screened on the basis of their accumulated FC values and VIP values. These differentially accumulated metabolites best represent the differences between Zhongzhe 1 sugarcane, which exhibits high resistance to smut disease, and G160 sugarcane, which exhibits low resistance to smut disease, and could provide reference data for future studies. Analysis of the metabolites that differentially accumulated between the Zhongzhe 1 sugarcane leaves and G160 sugarcane leaves showed that flavonoids were the main metabolites (Table 2), of which 56 differentially accumulated between Zhongzhe 1 sugarcane and G160 sugarcane (Figure 6). It is increasingly believed that flavonoids can promote the physiological survival of plants and protect them from fungal pathogens and environmental stress [18,19,20,29]. The abundance of 48 flavonoids, namely, 10 kaempferol, 1 apigenin and 3 catechin flavonoids, increased in Zhongzhe 1 sugarcane (Figure 6). Previous studies have suggested that kaempferol [30,31], chalcone [32] and catechin [33] are bioactive substances that have antioxidant, antibacterial and antifungal properties. It has also been reported that endophytic fungi can cause the accumulation of flavonoids in the leaves of *Cinnamomum longepaniculatum* [34]. In addition, the qRT–PCR results of the genes encoding key enzymes involved in the synthesis of flavonoids were consistent with the results of our clustering analysis of flavonoids in Zhongzhe 1 sugarcane and G160 sugarcane. Therefore, we believe that the flavonoids in Zhongzhe 1 sugarcane are related to the plant stress response and that the higher disease resistance of this variety may be associated with the accumulation of flavonoids.

LPE is a natural phospholipid that functions in plant innate immunity and early stages of plant senescence [22], and lyso-phosphatidylcholines (LPCs) are naturally occurring bioactive lipid molecules that significantly increase in abundance following pathogen inoculation [39]. Völz et al. [22] found that LPE treatment induced the expression of genes involved in the signaling and biosynthesis of the defense-related hormone salicylic acid (SA) and increased the resistance of LPE-pretreated *Arabidopsis* plants compared to mock-pretreated controls. The expression of pathogenesis-related (PR) genes (PR1-a, PR-3, and PR-4b) was found to strongly increase in LPC 18:1-treated tobacco leaves, indicating that LPC 18:1 may be involved in the pathogen defense response [39]. In the current study, a total of eleven LPEs and LPCs were SCMs; however, we discovered that nine of them, namely, four LPEs (LPE 18:3 (2n isomer)*, LPE18:3*, LPE 18:1 (2n isomer)* and LPE 18:1*) and five LPCs (LPC 16:2 (2n isomer), LPC 18:2 (2n isomer), LPC 18:1, LPC 18:1 (2n isomer) and LPC 20:3) highly accumulated in Zhongzhe 1 sugarcane. These findings indicate that the high resistance to smut disease of Zhongzhe 1 sugarcane may be related to lipids and that the defense mechanisms against smut disease in sugarcane may be associated with the lipid metabolism pathway.

Terpenoids and tannins are widely found in higher plants and comprise the most diverse group of secondary metabolites [40]. Terpenoids mainly include low-molecular-weight monoterpenes, sesquiterpenes, diterpenes and triterpene derivatives, which are widely found in plant tissues such as leaves, flowers and fruits [40,41]. The main functions of terpenoids and tannins include enhancing plant disease resistance and promoting plant resistance to natural enemies in direct and indirect manners [42]. In the present study, most terpenoids and tannins significantly highly accumulated in Zhongzhe 1 sugarcane, which exhibits high resistance to smut disease. The metabolites consisted of six terpenoids (-epoxydihydroartemisinic acid, sweroside, α-amyrenone, frehmaglutoside G, myricadoil and rutundic acid) and three tannins (ellagic acid-4-O-glucoside, geraniin* and geraniinic acid C*), whereas three terpenoids (colubrinic acid, hypoglycyrrhizic acid (β), zizyberanal acid) and one tannin significantly highly accumulated in G160 sugarcane, which exhibits low resistance to smut disease. Terpenoids and tannins are involved in resistance to diseases and pests, and several studies have shown that reducing tannin concentrations may have a negative effect on plant resistance [43,44]. Moreover, recent studies have proven that a variety of terpenoids, such as farnesene, can participate in the interaction of plants and biotic stress [45,46]. The increase in the concentrations of some terpenoids can improve the disease resistance of plants [47]. Therefore, we suggest that the terpenoids and tannins in Zhongzhe 1 sugarcane are associated with the plant response to pathogen stress and that their higher disease resistance may be associated with the accumulation of terpenoid and tannin compounds.

Phenolic acids and organic acids are some of the most important components involved in plant disease resistance, and the synthesis of phenolic compounds acts as the first defensive response against biotic stress and abiotic stress [23]. In this study, most phenolic acids and organic acids significantly decreased in accumulation in Zhongzhe 1 sugarcane, which exhibits high resistance to smut disease. Free phenolic acids and organic acids act as effective antifungal agents due to the formation of structural barriers [48]. The phenylpropanoid pathway is a rich source of plant metabolites, is indispensable for lignin biosynthesis, and is a starting point for the production of many other important compounds, such as flavonoids, coumarins and lignin [23]. The lower concentrations of phenolic acids and organic acids in Zhongzhe 1 sugarcane than in G160 sugarcane may occur because phenolic acids and organic acids are used to producing other compounds. These findings indicate that the defense mechanisms of Zhongzhe 1 sugarcane may not be associated with phenylpropanoid metabolism. Unlike studies on the defense mechanism of grape plants against powdery mildew, Yu’s study [49] found that the defense mechanism of grape varieties after powdery mildew infection may be related to phenylalanine metabolism. These findings suggested that different-resistant plants may have different defense mechanisms against microorganisms.

We also observed an interesting phenomenon in which most amino acids, nucleotides and their derivatives significantly increased in abundance in G160 sugarcane, which exhibits low resistance to smut disease. Amino acids are the raw materials for protein synthesis in plants and other organisms, and nucleotides are the basic constituents of DNA and RNA [50,51]. The higher concentration of amino acids, nucleotides and their derivatives in G160 sugarcane than in Zhongzhe 1 sugarcane may explain why pathogenic microorganisms are more amenable to infestation in the former, thus leading to disease susceptibility.

## 4. Materials and Methods

### 4.1. Plant Materials

The sugarcane varieties Zhongzhe 1 sugarcane and G160 sugarcane, which are offspring of the same parents (ROC25 and Yunzhe89-7), were used as test materials [8,9,10]. Zhongzhe 1 sugarcane, which is a member of the Zhongzhe series, is highly resistant to smut disease, but G160 sugarcane is highly susceptible to smut disease. All the plant materials used in this study were grown in the germplasm resources nursery at the State Key Laboratory for the Conservation and Utilization of Subtropical Agro-bioresources (Guangxi University). Leaf tissues of the sugarcane varieties Zhongzhe 1 sugarcane and G160 sugarcane were collected for metabolomic analysis, placed in liquid nitrogen and then stored at −80 °C. Total genomic DNA was extracted from young leaves according to the cetyl-trimethylammonium bromide (CTAB) method [52].

### 4.2. Root Tip Collection and Pretreatment

The roots were sampled at appropriate stages when the lengths of the roots were 1–2 cm. The samples were pretreated in an ice water bath for 24 h before they were transferred to Carnoy’s fixative fluid I (ethanol:glacial acetic acid mixture at 3:1 (*v*/*v*)) for 24 h and then ultimately to 70% ethanol, after which they were stored at −20 °C. After they were treated with 1% cellulase (Yakult, Tokyo, Japan) and 2% pectinase (Yakult, Japan) at 37 °C for 1 h, the root tips were cut into individual cells to facilitate observations of chromosome number and morphology. Slides containing cells clearly showing chromosomes in metaphase were dried and marked for subsequent experiments. The slides were prepared for GISH analysis through UV crosslinking (1250 mJ/cm^2^, 3 min), which resulted in the chromosomes being attached to the slides.

### 4.3. GISH Procedure

The GISH technique was performed as described previously by D’hont et al. [53], with moderate improvements. Genomic DNA from the female parent (ROC25) and male parent (Yunzhe89-7) labeled with DIG-11-dUTP (Roche, Basel, Switzerland) were used as probes for GISH analysis. After more than 16 h of hybridization in a dark and humid box at 37 °C, an anti-DIG fluorescein kit (Roche, Mannheim, Germany) was used to visualize the combined zone of the probes, and Vectashield H-1300 (Vector, Newark, CA, USA) was used to counterstain the chromosomes. The details of the procedures are available in the article by Pachakkil et al. [24]. The fluorescent signals were observed with a microscope (ZEISS Imager M2, Jena, Germany) and subsequently imaged (ZEISS ICc5, Germany).

### 4.4. LC–ESI–MS/MS Analysis and Differentially Accumulated Metabolite Identification

After freezing the leaf samples in liquid nitrogen, the sample extracts were analyzed using a UPLC–ESI–MS/MS system (ultra-performance liquid chromatograph, Shimadzu Nexera X2, Kyoto, Japan, www.shimadzu.com.cn/ accessed on 8 May 2021; mass spectrometer, Applied Biosystems 4500 Q TRAP, Waltham, MA, USA, www.appliedbiosystems.com.cn/ accessed on 8 May 2021). The detection was carried out as previously described [27]. The analytical conditions were as follows: for the ultra-performance liquid chromatograph, an Agilent SB-C18 column, Santa Clara, CA, USA (1.8 µm, 2.1 × 100 mm) was used. The mobile phase consisted of solvent A (pure water with 0.1% formic acid) and solvent B (acetonitrile with 0.1% formic acid). Sample measurements were performed by the use of a gradient program in which the starting conditions were 95% A and 5% B. Within 9 min, a linear increase to 5% A and 95% B began, and the composition of 5% A and 95% B was maintained for 1 min. Subsequently, a composition of 95% A and 5.0% B was adopted within 1.10 min and maintained for 2.9 min. The column oven was set to 40 °C, and the injection volume was 4 μL. The effluent was alternatively connected to an electrospray ionization (ESI) triple quadrupole linear ion trap (QTRAP)–mass spectrometer.

The concentrations of metabolites were identified based on previously described methods [28]. Specifically, metabolites were identified based on their parametric values (*m*/*z* data, retention time, and fragmentation partners) and compared with those of the contents of a self-constructed database (MetWare) and the Human Metabolome Database (HMDB) (http://www.hmdb.ca/, accessed on 8 May 2021), and annotation information was obtained. PCA and PLA-DA were conducted to identify the SCMs, criteria of which were a fold-change (FC) ≥ 2 or ≤0.5 and VIP ≥ 1. PCA and PLS-DA were implemented in R software (www.rproject.org/, accessed on 8 May 2021) to investigate variety-specific accumulation of metabolites in the leaves [30,54]. Finally, the SCMs between Zhongzhe 1 sugarcane and G160 sugarcane were analyzed via Kyoto Encyclopedia of Genes and Genomes (KEGG) pathway enrichment.

### 4.5. Quantitative Real-Time PCR

qRT–PCR was used to measure the differences in the relative expression of the genes encoding key enzymes involved in flavonoid synthesis (*IFS*, *F3H* and *DFR*) and 3 other important genes encoding defense-related enzymes (*SuChi*, *SuGluD1* and *SuPAL*) in Zhongzhe 1 sugarcane and G160 sugarcane. Total RNA was extracted by grinding 5~200 mg of leaf tissue to a powder in liquid nitrogen, and the total RNA concentration was determined according to the methods of Singh et al. [55]. cDNA was synthesized by reverse transcription of the total RNA according to the procedures of Singh et al. [55] and stored at −20 °C. The expression of the *IFS*, *F3H*, *DFR*, *SuChi*, *SuGluD1* and *SuPAL* genes was measured via qRT–PCR, with the β-tubulin gene used as an internal reference. Each reaction was repeated three times, and the results were analyzed by the 2^−ΔΔCt^ method. The reaction system was based on that in a study of Singh et al. [55], and the sequences of the primers used, which were synthesized by Bioengineering (Shanghai, China) Co., are shown in Table 2.

### 4.6. Statistical Analysis

All experiments were conducted in triplicates. Data were analyzed by using analysis of variance (ANOVA) with Duncan’s multiple range test (DMRT). Standard errors were calculated for all mean values, and differences were considered significant at the *p* ≤ 0.05 level.

## 5. Conclusions

Breeding for disease resistance is the most economical and effective measure to prevent and control sugarcane smut. The cytogenetic characteristics and metabolomic differences between the new sugarcane smut-resistant variety Zhongzhe 1 and the susceptible variety G160 improve our understanding of smut defense mechanisms. GISH analysis showed that the ploidy number of Zhongzhe 1 sugarcane and G160 sugarcane were 114 and 110, respectively; however, Zhongzhe 1 sugarcane and G160 sugarcane contain different numbers of chromosomes from the female parent (ROC25) and the male parent (Yunzhe89-7). Moreover, a total of 258 SCMs were identified in Zhongzhe 1 sugarcane with high resistance (HIII) to smut disease and G160 sugarcane with low resistance (LI): 56 flavonoids, 52 phenolic acids, 30 lipids, 26 organic acids, 26 amino acids and their derivatives, 19 nucleotides and derivatives, 5 alkaloids, 9 terpenoids, and 35 others. Multivariate statistics revealed a distinct difference in metabolic pathways between Zhongzhe 1 sugarcane and G160 sugarcane, and both had unique functional metabolites. Differences in chromosome composition may constitute the genetic basis of the differences in the resistance of Zhongzhe 1 sugarcane and G160 sugarcane to smut disease, and high accumulation of flavonoids, lipids, terpenoids and tannins may constitute the basis of the resistance to smut disease in Zhongzhe 1 sugarcane.

## Figures and Tables

**Table 1:** Classification of 258 SCMs.

Category	Phenolic Acids	Flavonoids	Lipids	Organic Acids	Amino Acids and Derivatives	Nucleotides and Derivatives	Alkaloids	Terpenoids	Others
Amounts in each category	118	138	98	73	73	38	17	33	113
Amounts of differentially accumulated metabolites	52	56	30	26	26	19	5	9	35
Percentages of differentially accumulated metabolites (%)	44.1	40.1	30.1	35.6	35.6	50.0	29.4	27.3	31.0

Note: The “Others” category includes stilbene, quinones, tannins, lignans and coumarins.

**Table 2:** Primers for qRT–PCR.

Gene	Upstream Primers (5′→3′)	Downstream Primers (5′→3′)	Reference
*IFS*	ACAACGGCGGAACATACG	ACACTGCTTGCCACTCACC	[35]
*F3H*	AAGAAGTGGAGCAAGGGAAAG	CGGAGACATTGGTGGAGAAA	[35]
*DFR*	ATGTTGCTACACCGTGGTTAC	AAATCGAAAGATCCCTCCTC	[35]
*SuChi*	ACGGCTACGGCGACAACA	GTCCGCTGACCAGATGAAGAG	[36]
*SuGluD1*	TGCTACTTCTTATCCACCCTCTG	CGTTGACATAGAAAGGTGAGCC	[37]
*SuPAL*	CTCGAGGAGAACATCAAGAC	GTGATGAGCTCCTTCTCG	[38]
*β-tubulin*	ATGTTCAGGCGCAAGGCTT	TCTGCAACCGGGTCATTCAT	[35]

## Data Availability

Not applicable.

## Supplementary Materials

The following supporting information can be downloaded at: https://www.mdpi.com/article/10.3390/plants12040810/s1, Table S1: A total of 701 metabolites were identified through positive and negative ionization modes; Table S2: A total of 258 metabolites were identified as significantly changed metabolites.
